# Atlanta Academy of Medicine

**Published:** 1872-02

**Authors:** 


Atlanta Academy of Medicine.—While we must apologize to a few of our readers who reside in the cities, and have advantages of medical societies, and are familiar with their discussions, for the large amount of space occasionally occupied by the transactions of the Atlanta Academy of Medicine, we are assured, at the same time, from various sources, that nothing contained in the Journal is more interesting to our village and country readers, who must, of course, make up the large mass of the profession in the Southern States, at least. We also believe that, with a considerable proportion of old and familiar material contained in the discussions and papers, something of special interest to all is being constantly furnished from this source.

